# Effect of weight change on the association between overall and source of carbohydrate intake and risk of metabolic syndrome: Tehran lipid and glucose study

**DOI:** 10.1186/s12986-023-00761-0

**Published:** 2023-09-12

**Authors:** Somayeh Hosseinpour-Niazi, Bahar Bakhshi, Parvin Mirmiran, Zahra Gaeini, Farzad Hadaegh, Fereidoun Azizi

**Affiliations:** 1 Nutrition and Endocrine Research Center, Research Institute for Endocrine Sciences, Shahid Beheshti University of Medical Sciences, Tehran, Iran; 2 School of Nutritional Sciences and Wellness, University of Arizona, Tucson, AZ USA; 3 Department of Clinical Nutrition and Dietetics, Faculty of Nutrition Sciences and Food Technology, National Nutrition and Food Technology Research Institute, Shahid Beheshti University of Medical Sciences, No. 24, A’rabi St., Yeman Av., P.O. Box: 19395-4763, Velenjak, Tehran Iran; 4 Prevention of Metabolic Disorders Research Center, Research Institute for Endocrine Sciences, Shahid Beheshti University of Medical Sciences, Tehran, Iran; 5 Endocrine Research Center, Research Institute for Endocrine Sciences, Shahid Beheshti University of Medical Sciences, Tehran, Iran

**Keywords:** Carbohydrates, Whole grains, Refined grains, Simple sugar, Weight change, Metabolic syndrome

## Abstract

**Background:**

In this prospective cohort study, we aimed to evaluate the association between dietary carbohydrates, whole grains, refined grains, and simple sugar with the risk of metabolic syndrome (MetS) and assess the effect of weight change on these associations.

**Methods:**

This study was conducted within the framework of the Tehran Lipid and Glucose Study (TLGS). We included 1915 healthy participants with complete demographic, anthropometric and dietary measurements, among whom 591 developed MetS during 8.9 years of follow-up. Intake of dietary carbohydrates, whole grains, refined grains, and simple sugar was assessed with a validated semi-quantitative food frequency questionnaire. Multivariable adjusted Cox regression was used to estimate hazard ratios (HRs) for MetS events across tertiles of dietary variables. Using joint classification, the effect of weight change on the association between dietary variables and risk of MetS was assessed by Cox regression.

**Results:**

Carbohydrate intake was not associated with the risk of MetS in multivariable-adjusted models. Whole grains intake was inversely associated with the risk of MetS (HR: 0.78, CI: 0.63–0.98), while this association disappeared after adjustment for weight change. The risk of MetS increased by 11% (1.11, 1.09–1.14) for each 3% energy increment from simple sugar, and by 5% (1.05, 1.03–1.08) for each 1 serving/day increment in refined grains. Consumption of refined grains increased the risk of MetS; an effect modification of this association was found by weight change. Among subjects with weight loss, intake of refined grains < median intake decreased the risk of MetS (0.59, 0.41–0.87). However, consumption of refined grains ≥ median intake increased the risk of MetS in individuals with weight gain (1.47, 1.08–2.01). Simple sugar was positively associated with an increased risk of MetS, after adjustment for weight change (3.00, 2.37–3.82). In joint classification, intake of simple sugar greater than median intake increased the risk of MetS, independent of weight change.

**Conclusion:**

Our findings suggest an effect modification by weight change on the association between carbohydrates, and refined grains intake and the risk of MetS. Weight loss along with lower consumption of carbohydrates, and refined grains reduced the risk of MetS. However, simple sugar intake, regardless of weight change, was associated with an increased risk of MetS.

## Introduction

Dietary carbohydrate consumption has been at the forefront of population-level nutrition recommendations to prevent and manage diet-related chronic diseases [1] and has varying impacts on postprandial glucose metabolism [2]. Although it is well established that both carbohydrate quantity and quality affect chronic disease risk [3], epidemiological studies have demonstrated conflicting results concerning total and specific dietary carbohydrates and cardiometabolic health [4–6]. The Atherosclerosis Risk in Communities (ARIC) and the Prospective Urban Rural Epidemiology (PURE) studies, two large prospective cohorts, have indicated a U-shaped effect of energy from carbohydrates intake and mortality, suggesting that both low and high carbohydrate intake was associated with an increased risk of mortality [7, 8]. Recently, an updated systematic review and meta-analysis of 18 observational studies reported that the highest versus lowest categories of carbohydrates intake were not associated with the risk of type 2 diabetes mellitus (T2DM). However, this meta-analysis indicated a j-shaped dose-response association was observed, wherein the risk of T2DM considerably increased at 70% energy from carbohydrate intake [9]. Specific dietary carbohydrates, including whole grains, refined grains, and simple sugar, have been commonly used to measure carbohydrate quality with distinct functional properties and health benefits [10]. Traditionally, evidence from systematic reviews and meta-analysis of observational studies and human trials suggest that whole grains are inversely and refined grains are positively associated with the cardiometabolic outcomes [6, 10–12]. However, inconsistencies exit regarding the direction and magnitude of association between simple sugar intake and the risk of T2DM [13–15].

Metabolic Syndrome (MetS), defined as the constellation of interrelated metabolic abnormalities, predisposes individuals to a substantially higher risk of T2DM and cardiovascular disease (CVD) [16]. Lifestyle modifications, including therapeutic dietary strategies, weight management, and physical activity interventions, have been the cornerstones of MetS prevention and management [17]. With regards to the effect of dietary carbohydrate intake on MetS, a dose-response meta-analysis of observational studies concluded a weak linear association between carbohydrate consumption and MetS [4]. However, it should be noted that in this meta-analysis, heterogeneity between the studies was high (*I2* = 82.0%, P = 0.000), warranting future prospective studies to confirm these results [4].

Weight gain is a risk factor for MetS development and progression [18]. In our previous studies, we reported the effect modification of weight change on the association between dietary food groups such as fruits, vegetables, fruit juice and risk of MetS [19, 20]. However, other studies have shown that restriction of carbohydrate intake decreased [21], and sugar-sweetened beverages (SSBs) increased the risk of the MetS [20], independent of weight changes. The extent to which weight change can modulate the association between consuming carbohydrates, refined grains, whole grains, and simple sugar and metabolic syndrome remains unknown [22]. Therefore, in the present prospective cohort study, we aimed to (1) evaluate the association between dietary carbohydrates, whole grains, refined grains, and simple sugar intake and the risk of MetS, and (2) assess the effect of weight change (weight loss, stable weight, or weight gain) on the observed associations.

## Materials and methods

### Study population

We conducted this prospective population-based study within the framework of Tehran Lipid and Glucose Study (TLGS), which is an ongoing prospective study to prevent non-communicable diseases. The details of this study have been provided elsewhere [23]. In our first survey, initiated in March 1999, more than 15,000 individuals aged ≥ 3 years were enrolled from district 13 of Tehran, the capital of Iran, using multistage stratified cluster random sampling. The population of district 13 represents the urban population of Tehran. Since 1999, the participants of TLGS underwent assessments for sociodemographic factors, lifestyle, medication use, socioeconomic status, anthropometric indices, and medical history of cardiovascular risk factors. Information was documented every three years in face-to-face visits by the local research team to update the previous data. Phases II, III, IV, V, and VI were prospective follow-up studies conducted during 2002–2004, 2005–2008, 2008–2011, 2012–2015, and 2016–2018, respectively. In the current study, because of the small sample size for dietary assessment in Phases I and II of the research and using 24-hour dietary recalls, baseline examination data was utilized from phase III of the TLGS (2006–2008). We used the baseline examination data from phase III of TLGS (2005–2008) and followed up the participants up to phase VI of TLGS (2016–2018) in an 8.91-year follow-up (IQR: 7.98–9.69). In the third survey of TLGS (2005–2008), medical history and physical examination were collected for 12,523 participants, after which a representative sample of 4920 participants was randomly selected based on their age and gender to complete further dietary assessment. Of 4920 participants, 3462 agreed to complete a food frequency questionnaire (FFQ). The characteristics of participants who completed the FFQ were similar to those of the total population in phase III of TLGS [24]. Of 3462 participants, 3265 adults aged 19–74 years with complete information were selected from phase III of TLGS (2005–2008), while the following samples were excluded: (1) individuals with MetS at baseline (n = 879); (2) pregnant or lactating women at baseline or follow-up (n = 28); (3) subjects with daily energy intake < 500 and > 4000 kcal per day (n = 115) [25]; (4) subjects with any specific diets as a result of their hyperlipidemia, hypertension, and hyperglycemia (n = 26); and (5) subjects with missing laboratory or anthropometric data related to the diagnosis of MetS during the follow-up (n = 309). The final analysis was conducted on 1915 participants until 2018, with a response rate of 66% during an 8.9-year follow-up period (IQR: 7.98–9.69).

The study protocol was approved by the Ethics Committee of the Research Institute for Endocrine Sciences (RIES) of Shahid Beheshti University of Medical Sciences, Tehran, Iran. Written informed consent was also obtained from all participants.

### Anthropometric measurements

Weight was measured using a digital scale (Seca 707; range: 0-150 kg; Seca GmbH, Germany), in the fasted state, with minimal clothing, without shoes, and recorded to the nearest 100 g. Height was also measured in a standing position, with shoulders in neutral alignment without shoes, using a stadiometer (Seca 225; Seca GmbH, Germany), and recorded to the nearest 0.5 cm. The body mass index (BMI) was calculated by dividing weight in kilograms by height in meters squared. Moreover, waist circumference (WC) was measured at the umbilical level using an un-stretched tape measure (accuracy, 0.5 cm). After a 15-minute rest, blood pressure was measured using a standardized mercury sphygmomanometer (calibrated by the Iranian Institute of Standards and Industrial Research) on the right arm twice, at least 30 s apart. The average of the two measurements was reported as the subject’s blood pressure.

### Assessment of other variables

At baseline, general characteristics of the participants, including demographic, lifestyle (smoking status and physical activity), socioeconomic status (education and employment), medication regimen (e.g., antihypertensive, lipid-lowering, and anti-diabetes drugs), and medical history were collected by trained researchers, using a standardized questionnaire. Physical activity was also assessed using the Modifiable Activity Questionnaire (MAQ), and the frequency and amount of time spent per week on physical activity over the last year were recorded [26]. The physical activity levels were expressed as metabolic-equivalent (MET) hours per week (MET-h/week) [27]. The reliability and convergent validity of the Persian version of MAQ have been reported elsewhere [28].

### Dietary assessment

During face-to-face interviews with expert dietitians, a validated semi-quantitative FFQ was used to determine the frequency of each food item daily, weekly, or monthly in the past year. The portions were converted to grams according to a standard unit or portion size. Iranian food composition table (FCT) was used to calculate macro- and micronutrients [29].

Of 1915 participants at baseline, 592 completed all four FFQs, 804 completed three FFQs, 316 completed two FFQs, and 203 refused to complete any FFQs during the follow-up time. The last observation carried forward method was also used to impute the missing values [30]. In the present study, due to the crucial effect of recent dietary intakes on the association between diet and chronic disease, we used an alternative approach according to the Hu et al. formula [30]. This approach adds more weight to the recent dietary assessments, aiming to reduce within-subject variability and evaluate the long-term diet more concisely.

Whole grains included Iranian bread of Sangak, Barbari, taftoon, toasted bread (whole grain), popcorn, cooked barley, bulgur, corn, and biscuits prepared with whole grains. Refined grains included Iranian bread lavash, baguette, pasta, rice, reshte, wheat flour, and noodles. Intake of refined grains and whole grains was evaluated by adjusting the total energy intake according to residual model [25].

Simple sugar was the percentage of calories from table sugar, honey, jam, Gaz, Sohan, and Noghl, and cubed sugar. For carbohydrates, refined grains, and whole grains, a good correlation coefficient existed between FFQ and multiple 24 recalls and between two FFQs [31]. Moreover, the dietary patterns’ reliability, validity, and stability were reasonable based on the data collected from the FFQ over eight years [32].

### Biochemical assessment

For biochemical measurements, after 12–14 h of overnight fasting, venous blood samples were collected in vacutainer tubes and centrifuged within 30–45 min of collection for all subjects. The fasting plasma glucose (FPG), high-density lipoprotein-cholesterol (HDL-C), and triglyceride (TG) levels were measured in the TLGS research laboratory on the day of sample collection, using a Selectra 2 autoanalyzer (Vital Scientific, Spankeren, the Netherlands) and commercial kits (Pars Azmoon Inc., Tehran, Iran). FPG level was measured using an enzymatic colorimetric method with the glucose oxidase technique. The inter- and intra-assay coefficients of variation (CV) at baseline and after follow-up were both below 2.3%. TG was also assayed using an enzymatic colorimetric method with glycerol phosphate oxidase. HDL-C was measured after the precipitation of apolipoprotein B-containing lipoproteins with phosphotungstic acid. In all baseline and follow-up assays, intra- and inter-assay CVs were below 2.1% and 3.0% for TG and HDL-C. All samples were analyzed when the internal quality control met the acceptable criteria.

### Definition of MetS

According to the Joint Interim Statement, diagnosis of MetS requires the presence of three or more of the following criteria [16]: (1) elevated glucose concentration (FPG ≥ 100 mg/dL) or treatment with anti-hyperglycemic medications; (2) elevated serum TG concentration (≥ 150 mg/dL) or treatment with anti-hypertriglyceridemia medications; (3) reduced serum HDL-C concentration (< 50 mg/dL in women and < 40 mg/dL in men); (4) elevated blood pressure (≥ 130/85 mmHg) or treatment with anti-hypertensive medications; and (5) enlarged abdominal circumference (≥ 95 cm according to the population- and country-specific cut-off points for Iranian adults of both genders) [33].

### Definition of weight change

Weight change was calculated by subtracting the baseline weight from the follow-up one (phase IV) and multiplying it by 100. Participants were categorized as those who lost weight (> 3%), those with weight stability (± 3%), and those who gained weight (> 3%) [34].

### Statistical analysis

Data are reported as mean (SD) and median (25th and 75th percentiles) for continuous variables or percentage for categorical variables. An alternative approach was used to evaluate the consumption of carbohydrates, whole grains, refined grains and simple sugar during the 8.9-year follow-up [30]. Dietary carbohydrate, whole grains, refined grains, and simple sugar intake was categorized into tertiles. Baseline characteristics and energy-adjusted dietary variables were described across the tertiles of dietary carbohydrates, whole grains, refined grains, and simple sugar using the general linear model and Chi-square test for continuous and categorical variables, respectively. Moreover, Cox proportional-hazards regression models were used to estimate the hazard ratios (HRs) and their 95% confidence intervals (CIs) for the incidence of MetS and weight change (< 3% vs. ≥ 3%) across the tertiles of dietary carbohydrates, whole grains, refined grains, and simple sugar intake. Moreover, HRs (95% CI) for the MetS and weight gain was estimated per 5% energy increment for carbohydrate, 3% energy increment for simple sugar, and 1 serving/day increment for both whole grains and refined grains. The first model was a univariate analysis (model 1), while the second model was adjusted for potential confounders, including age, sex, smoking status, physical activity, total energy intake, dietary fat, dietary protein, healthy eating index (HEI) [35], family history of diabetes, and history of CVD. The third model was further adjusted for weight change. The linearity of trends was determined by integrating the median values of tertiles as continuous variables into the Cox regression models.

Among carbohydrate intake and its sources, carbohydrate (*P* value = 0.042), whole grains (*P* value = 0.043), and simple sugar (*P* interaction = 0.038) had significant interactions with weight change on the risk of MetS in the multivariable model; although interaction test was tended to be significant for refined grains (*P* interaction = 0.071). Therefore, we evaluated the effect of weight change on the association between dietary variables and the risk of MetS. Based on the multivariable Cox regression model, by joint classification, we estimated the HRs and 95% CIs for Mets, according to the weight change during the follow-up (> 3%, ± 3%, or, < 3%) [34]. Subjects with weight stability and consumption of carbohydrates, refined grains, whole grains, and simple sugar, lower than the median intake, were considered as references.

To correct for multiple testing, false discovery rate q-values were computed from *P* values using the Benjamini–Hochberg procedure [36]. All statistical analyses were performed in SPSS version 15.0 (SPSS Inc., Chicago, IL, USA), and *P*-values less than 0.05 were considered statistically significant.

## Results

The baseline mean (SD) age and BMI of the participants (male, 40.5%) were 36.5 (13.3) years and 25.6 (4.5) kg/m^2^, respectively. The median (interquartile range) intakes of dietary variables were: carbohydrate 53.1% of total energy (46.0-58.6); whole grains 4.0 serving/day (2.4–6.3); refined grains 6.6 serving/day (3.9–8.9); and simple sugar 15.7% of total energy (8.0-15.9).

We documented 591 new cases of MetS during the median follow-up of 8.91 years (IQR: 7.98–9.69). The baseline characteristics of the participants according to tertiles of carbohydrate, and refined grains are presented in Table 1. Compared with individuals at the lowest tertile of intake of carbohydrate and refined grains, those at the highest tertile were statistically significantly older, were smokers, had a higher BMI at baseline, experienced more weight change during the follow-up, and had a significantly higher intake of total energy, carbohydrate and fiber and a lower intake of fat and cholesterol. A significant reduction in HEI score was observed across the carbohydrate and refined grains tertiles.

**Table 1:** Baseline characteristics of participants across tertiles of carbohydrate, and refined grains: Tehran Lipid and Glucose Study

	Carbohydrate, % of calorie				Refined grains, serving/day			
	T1	T2	T3	P value	T1	T2	T3	P value
Median intake	44.4	53.1	60.2		2.9	6.7	9.9	
Range of intake	≤ 47.5	47.6–56.9	≥ 57.0		≤ 5.4	5.5–8.3	≥ 8.4	
n/N	186/640	179/637	226/638		138/638	176/639	277/638	
Continuous variables, Mean ± SE								
Age at baseline (y)	35.8 ± 0.5	36.0 ± 0.5	37.7 ± 0.5	0.021	35.1 ± 0.5	36.1 ± 0.5	38.2 ± 0.5	< 0.001
Physical activity (MET hour-week)	4.7 ± 0.3	5.2 ± 0.3	5.1 ± 0.3	0.290	4.6 ± 0.3	5.0 ± 0.3	5.4 ± 0.3	0.090
BMI at baseline (kg/m2)	25.1 ± 0.2	25.7 ± 0.2	25.9 ± 0.2	0.011	25.3 ± 0.2	25.1 ± 0.2	26.3 ± 0.2	< 0.001
Weight change (kg)	1.3 ± 0.5	3.2 ± 0.7	5.2 ± 0.8	0.012	2.4 ± 0.7	2.9 ± 0.7	6.3 ± 0.7	< 0.001
Categorical variables, number (%)								
Female	349 (54.5)	417 (65.5)	374 (58.6)	< 0.001	501 (78.4)	323 (50.6)	316 (49.5)	< 0.001
Smoker at baseline	126 (19.7)	130 (20.4)	160 (25.0)	0.046	98 (15.3)	152 (23.8)	166 (26.0)	< 0.001
Academic degrees	178 (27.8)	168 (26.4)	151 (23.7)	0.229	173 (27.1)	161 (25.2)	163 (25.5)	0.725
Occupational status, employed	286 (44.7)	251 (39.4)	289 (45.3)	0.065	432 (67.6)	348 (54.5)	309 (48.4)	< 0.001
Family history of diabetes	199 (31.1)	197 (30.9)	224 (35.1)	0.430	229 (35.8)	182 (28.5)	209 (32.8)	0.044
***Dietary intake***, Mean ± SE								
Total energy (Kcal/d)	2240 ± 35	2352 ± 35	2405 ± 35	0.004	2080 ± 34	2329 ± 34	2589 ± 34	< 0.001
Carbohydrate (% of total energy)	44.0 ± 0.2	52.5 ± 0.2	63.2 ± 0.2	< 0.001	53.4 ± 0.3	56.5 ± 0.3	59.7 ± 0.3	0.007
Protein (% of total energy)	14.9 ± 0.3	14.5 ± 0.3	14.5 ± 0.3	0.613	15.4 ± 0.3	14.5 ± 0.3	14.1 ± 0.3	0.009
Fat (% of total energy)	33.8 ± 0.2	30.1 ± 0.2	29.8 ± 0.2	< 0.001	33.7 ± 0.2	32.7 ± 0.2	30.3 ± 0.2	0.034
Total fiber (g/d)	35.7 ± 0.7	41.5 ± 0.7	42.2 ± 0.7	< 0.001	38.0 ± 0.7	41.4 ± 0.7	47.1 ± 0.7	< 0.001
Cholesterol (g/d)	230 ± 8.1	249 ± 8.2	217 ± 8.2	0.021	244 ± 8	236 ± 8	216 ± 8	0.052
Healthy eating index	69.6 ± 0.3	68.1 ± 0.3	67.6 ± 0.3	< 0.001	60.2 ± 0.2	69.4 ± 0.2	57.8 ± 0.2	< 0.001

n/N: Number of MetS /number of subjects; MET, metabolic equivalent; BMI, body mass index

The percentage of women and men increased from the first to the third tertile of carbohydrates and refined grains, respectively. Moreover, participants in the highest tertiles of refined grains were less employed, had less family history of diabetes, and had a lower intake of protein.

Table 2 presents the baseline characteristics of the participant across tertiles of simple sugar, and whole grains intake. Individuals in the third tertile of simple sugar were older, had higher BMI, higher weight gain during the follow-up, and had a significantly higher intake of total energy, fat, and fiber. Participants in the top tertile of the whole grains were mostly women, had higher physical activity levels, lower weight gain during the follow-up, consumed more energy, carbohydrate, and fiber, and less fat and cholesterol. With an increasing intake of whole grains, the HEI score increased however, the HEI score decreased across tertiles of simple sugar.

**Table 2:** Baseline characteristics of participants across tertiles of simple sugar and whole grains: Tehran Lipid and Glucose Study

	simple sugar, % of calorie				whole grains, serving/day			
	T1	T2	T3	*P* value	T1	T2	T3	*P* value
Median intake	6.4	15.7	16.7		2.0	4.0	7.4	
Range of intake	≤ 12.8	12.9–15.8	≥ 15.9		≤ 3.0	3.1–5.3	≥ 5.4	
n/N	93/639	247/667	251/609		235/639	181/638	175/638	
Continuous variables, Mean ± SE								
Age at baseline (y)	35.2 ± 0.5	36.7 ± 0.5	37.6 ± 0.5	0.006	37.2 ± 0.5	36.1 ± 0.5	36.1 ± 0.5	0.248
Physical activity (MET hour-week)	5.1 ± 0.3	5.1 ± 0.3	4.8 ± 0.3	0.743	4.5 ± 0.3	5.2 ± 0.3	5.3 ± 0.3	0.042
BMI at baseline (kg/m2)	25.1 ± 0.2	25.7 ± 0.2	26.0 ± 0.2	0.002	25.7 ± 0.2	25.5 ± 0.2	25.4 ± 0.2	0.465
Weight change (kg)	2.1 ± 0.7	4.8 ± 0.7	4.8 ± 0.7	0.015	6.9 ± 0.7	4.2 ± 0.7	1.6 ± 0.7	< 0.001
Categorical variables, number (%)								
Female	404 (63.2)	386 (57.9)	350 (57.5)	0.065	282 (44.1)	394 (61.8)	464 (72.7)	< 0.001
Smoker at baseline	119 (18.6)	158 (23.7)	139 (22.8)	0.062	150 (23.5)	133 (20.8)	133 (20.8)	0.421
Academic degrees	162 (25.4)	179 (26.8)	156 (25.6)	0.808	173 (27.1)	167 (26.2)	497 (26.0)	0.596
Occupational status, employed	381 (59.6)	374 (56.1)	334 (54.8)	0.205	337 (52.7)	272 (42.6)	217 (34.0)	< 0.001
Family history of diabetes	205 (32.1)	213 (31.9)	202 (33.2)	0.793	210 (32.9)	199 (31.2)	620 (32.4)	0.682
***Dietary intake***, Mean ± SE								
Total energy (Kcal/d)	2285 ± 35	2356 ± 35	2455 ± 36	0.004	2093 ± 34	2277 ± 34	2326 ± 34	< 0.001
Carbohydrate (% of total energy)	53.4 ± 0.3	53.3 ± 0.3	52.9 ± 0.3	0.564	53.0 ± 0.3	55.3 ± 0.3	57.3 ± 0.3	0.028
Protein (% of total energy)	14.4 ± 0.3	14.6 ± 0.3	14.9 ± 0.3	0.470	14.7 ± 0.3	15.2 ± 0.3	14.2 ± 0.3	0.062
Fat (% of total energy)	30.6 ± 0.2	32.0 ± 0.2	33.6 ± 0.2	0.005	31.1 ± 0.2	29.8 ± 0.2	27.8 ± 0.2	< 0.001
Total fiber (g/d)	40.8 ± 0.7	42.3 ± 0.7	43.4 ± 0.7	0.039	34.7 ± 0.6	40.7 ± 0.6	41.0 ± 0.6	< 0.001
Cholesterol (g/d)	227 ± 8	234 ± 8	235 ± 8	0.727	249 ± 8	238 ± 8	209 ± 8	0.002
Healthy eating index	61.1 ± 0.2	60.0 ± 0.2	59.3 ± 0.2	< 0.001	67.5 ± 0.2	60.0 ± 0.2	69.9 ± 0.2	< 0.001

n/N: Number of MetS /number of subjects; MET, metabolic equivalent; BMI, body mass index

Multivariable-adjusted HRs (95% CI) for MetS according to tertiles of carbohydrates, whole grains, refined grains, and simple sugar are presented in Table 3. The highest tertile of total carbohydrate intake was associated with an increase in the risk of MetS in model 1 (1.30: 1.07–1.57, *P* trend 0.007). Adjustment for confounding factors in models 2 and 3 diminished the association to non-significant levels (1.26: 0.97–1.58, *P* trend 0.043; 1.23: 0.88–1.47, *P* trend 0.089). The consumption of whole grain in the crude model (model 1) and after adjustment for confounders in model 2 was negatively associated with the risk of MetS (model 1: 0.73: 0.60–0.89, *P* trend 0.001; model 2, 0.78: 0.63–0.98, *P* trend 0.004)), although this association did not remain significant after adjustment for weight change. Both refined grains (model 1: 1.52: 1.24–1.85, *P* trend < 0.001; Model 2: 1.73: 1.43–2.12, *P* trend < 0.001; Model 3, 1.59, 1.31–1.93, *P* trend < 0.001) and simple sugar (model 1: 2.32: 2.62–4.21, *P* trend < 0.001; Model 2, 3.20: 2.53–4.07, *P* trend < 0.001; Model 3: 3.00, 2.37–3.82, *P* trend < 0.001) were positively associated with MetS risk in all three models. Furthermore, when carbohydrate intake and its source were considered as continuous variables, the risk of MetS increased by 11% (1.11, 1.09–1.14) for each 3% energy increment from simple sugar, and by 5% (1.05, 1.03–1.08) for each 1 serving/day increment in refined grains, in model 3.

**Table 3:** Multivariable adjusted hazard ratio (95% confidence interval) for metabolic syndrome across tertiles of carbohydrate, whole grains, refined grains and simple sugar: Tehran Lipid and Glucose Study

	Tertiles of intakes						
Variable	T1	T2	T3	*P*_trend_	Q value	Continuous variable	*P* value
Total carbohydrate (% of calorie)							
Median intake	44.4	53.1	60.2			Per 5% energy increment	
Range of intake	≤ 47.5	47.6–56.9	≥ 57.0				
Model 1	1	0.99 (0.80–1.21)	1.30 (1.07–1.57)	0.007	0.009	1.05 (1.01–1.10)	0.025
Model 2	1	1.03 (0.84–1.24)	1.26 (0.97–1.58)	0.043	0.051	1.04 (1.00-1.08)	0.047
Model 3	1	1.04 (0.86–1.26)	1.23 (0.88–1.47)	0.089	0.089	1.02 (0.97–1.06)	0.148
Whole grains (serving/day)							
Median intake	2.0	4.0	7.4			Each 1 serving/day increment	
Range of intake	≤ 3.0	3.1–5.3	≥ 5.4				
Model 1	1	0.72 (0.60–0.88)	0.73 (0.60–0.89)	0.001	0.002	0.96 (0.93–0.99)	0.010
Model 2	1	0.79 (0.66–0.98)	0.78 (0.63–0.98)	0.004	0.006	0.97 (0.94–1.01)	0.071
Model 3	1	0.85 (0.73–1.08)	0.87 (0.71–1.09)	0.076	0.082	0.98 (0.95–1.02)	0.192
Refined grains (serving/day)							
Median intake	2.9	6.7	9.9			Each 1 serving/day increment	
Range of intake	≤ 5.4	5.5–8.3	≥ 8.4				
Model 1	1	1.14 (0.92–1.40)	1.52 (1.24–1.85)	< 0.001	0.002	1.08 (1.06–1.10)	< 0.001
Model 2	1	1.15 (0.93–1.41)	1.73 (1.43–2.12)	< 0.001	0.002	1.07 (1.04–1.09)	< 0.001
Model 3	1	1.11 (0.91–1.37)	1.59 (1.31–1.93)	< 0.001	0.002	1.05 (1.03–1.08)	< 0.001
Simple sugar (% of calorie)							
Median intake	6.4	13.7	16.7			Per 3% energy increment	
Range of intake	≤ 12.8	12.9–15.8	≥ 15.9				
Model 1	1	2.92 (2.30–3.71)	2.32 (2.62–4.21)	< 0.001	0.002	1.14 (1.11–1.16)	< 0.001
Model 2	1	2.84 (2.23–3.61)	3.20 (2.53–4.07)	< 0.001	0.002	1.12 (1.10–1.15)	< 0.001
Model 3	1	2.67 (2.00-3.39)	3.00 (2.37–3.82)	< 0.001	0.002	1.11 (1.09–1.14)	< 0.001

Model 1 was crude

Model 2 was adjusted for age, sex, smoking status, physical activity, total energy intake, dietary fat, dietary protein, healthy eating index score, family history of diabetes, and history of cardiovascular disease (all variable that adjusted was at baseline)

Model 3 was additionally adjusted for weight change

Multivariable-adjusted HRs (95% CI) for weight change according to tertiles of carbohydrates, whole grains, refined grains, and simple sugar are presented in Table 4. Carbohydrates, refined grains, and simple sugar were associated with an increased risk of weight gain after adjustment for confounders; the corresponding HRs were 1.24 (1.02–1.51), 1.32 (1.09–1.65), and 2.75 (2.15–3.53), respectively. The consumption of whole grain was negatively associated with weight gain after adjustment for confouders (0.44: 0.34–0.48, *P* trend < 0.004). Furthermore, when carbohydrate intake and its source were considered as continuous variables, the risk of MetS increased by 12% (1.12, 1.10–1.15) for each 3% energy increment from simple sugar, and by 7% (1.07, 1.04–1.09) for each 1 serving/day increment in refined grains, in model 2.

**Table 4:** Multivariable adjusted hazard ratio (95% confidence interval) for weight change (< 3% vs. ≥ 3%) across tertiles of carbohydrate, whole grains, refined grains and simple sugar: Tehran Lipid and Glucose Study

	Tertiles of intakes						
Variable	T1	T2	T3	P _trend_	Q value	Continuous variable	P value
Total carbohydrate (% of calorie)							
Median intake	44.4	53.1	60.2			Per 5% energy increment	
Range of intake	≤ 47.5	47.6–56.9	≥ 57.0				
Model 1	1	0.99 (0.80–1.21)	1.30 (1.07–1.57)	0.007	0.008	1.05 (1.01–1.10)	0.025
Model 2	1	1.11 (0.89–1.37)	1.24 (1.02–1.51)	0.025	0.025	1.03 (0.99–1.08)	0.061
Whole grains (serving/day)							
Median intake	2.0	4.0	7.4			Each 1 serving/day increment	
Range of intake	≤ 3.0	3.1–5.3	≥ 5.4				
Model 1	1	0.64 (0.51–0.79)	0.49 (0.36–0.55)	< 0.001	0.002	0.96 (0.93–0.99)	0.010
Model 2	1	0.58 (0.48–0.70)	0.44 (0.34–0.48)	< 0.001	0.002	0.97 (0.94-1.00)	0.071
Refined grains (serving/day)							
Median intake	2.9	6.7	9.9			Each 1 serving/day increment	
Range of intake	≤ 5.4	5.5–8.3	≥ 8.4				
Model 1	1	1.25 (1.06–1.51)	1.38 (1.14–1.68)	< 0.001	0.002	1.08 (1.06–1.10)	< 0.001
Model 2	1	1.19 (0.98–1.47)	1.32 (1.09–1.65)	0.006	0.008	1.07 (1.04–1.09)	< 0.001
Simple sugar (% of calorie)							
Median intake	6.4	13.7	16.7			Per 3% energy increment	
Range of intake	≤ 12.8	12.9–15.8	≥ 15.9				
Model 1	1	2.92 (2.30–3.71)	3.32 (2.62–4.21)	< 0.001	0.002	1.14 (1.11–1.16)	< 0.001
Model 2	1	2.51 (1.96–3.21)	2.75 (2.15–3.53)	< 0.001	0.002	1.12 (1.10–1.15)	< 0.001

Model 1 was crude

Model 2 was adjusted for age, sex, smoking status, physical activity, total energy intake, dietary fat, dietary protein, healthy eating index score, family history of diabetes, and history of cardiovascular disease (all variable that adjusted was at baseline)

**Fig. 1:**
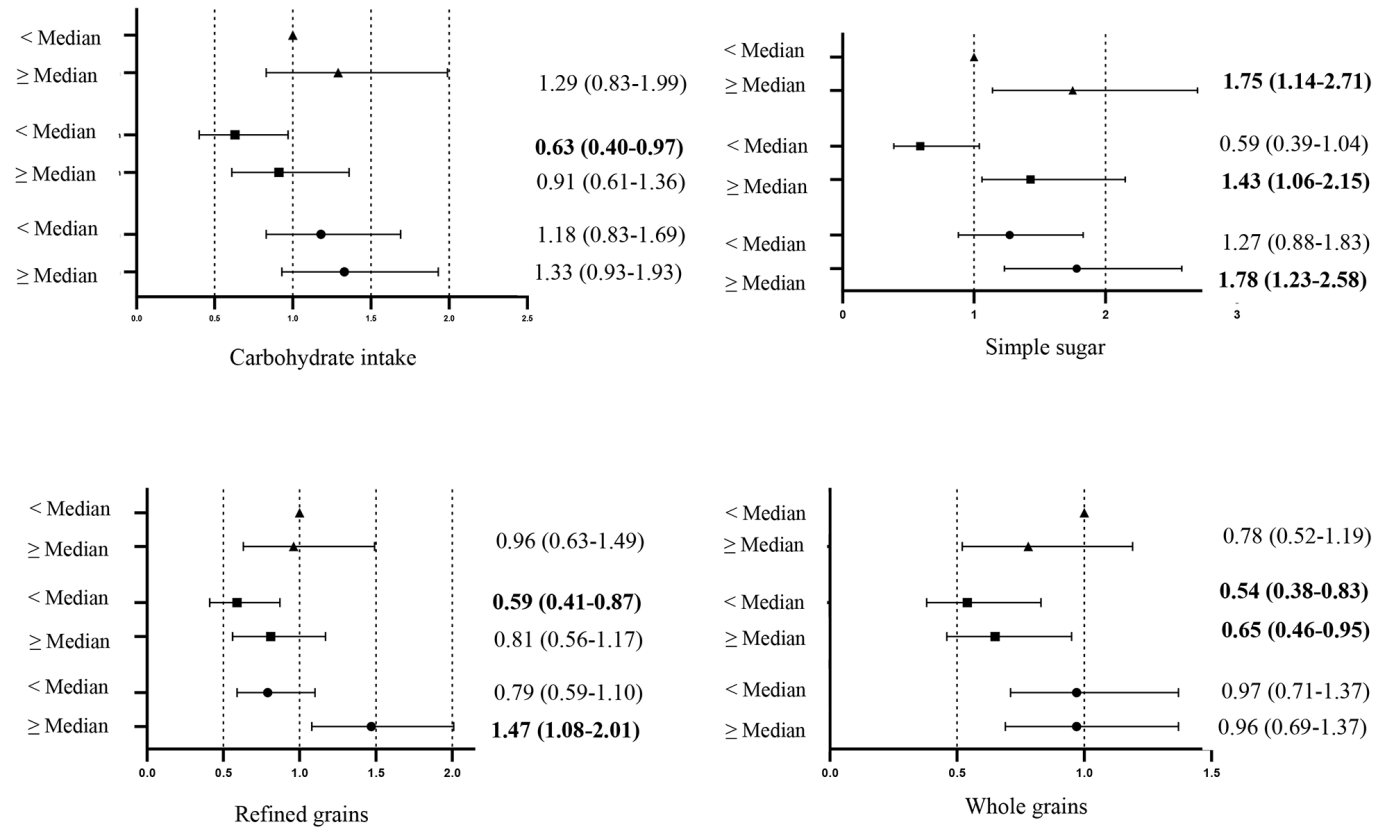
Hazard ratios of the combined effect of dietary carbohydrate, whole grains, refined grains and simple sugar (< median vs. ≥ median) and weight change (triangle, weight stable (± 3%); square, > 3% weight loss; and circle, > 3% weight gain) on risk MetS after adjustment for age, sex, smoking status, physical activity, total energy intake, dietary fat, dietary protein, healthy eating index, family history of diabetes, and history of cardiovascular disease. The median (interquartile range) intakes of dietary variables were: carbohydrate 53.1% of total energy (46.0-58.6); whole grains 4.0 serving/day (2.4–6.3); refined grains 6.7 serving/day (3.9–8.9); and simple sugar 15.7% of total energy (8.0-15.9)

Figure 1 presents multivariable-adjusted HRs (95% CIs) of MetS according to joint categories of carbohydrates, whole grains, refined grains, a simple sugar, and weight change. An effect modification by weight change on the association between carbohydrates, and refined grains intake and the risk of MetS was found. Consumption of carbohydrate and refined intake, < median was associated with a reduction in the risk of MetS in individuals who experienced weight loss (0.63: 0.40–0.97 for carbohydrate; 0.59, 0.41–0.87 for simple sugar). However, in subjects with weight gain, consumption of refined grains, ≥ median intake, increased the risk of MetS by 47% (1.47, 1.08–2.01). Consumption of simple sugar, ≥ median intake, was positively associated with the risk of MetS, independent of weight change. Moreover, weight loss protected against the risk of MetS independent of whole grains intake.

## Discussion

In this prospective cohort study, no associations between carbohydrate intake and MetS risk were identified. We found that whole grain intake was negatively associated with the risk of MetS after adjustment for confounding factors. However, the latter association disappeared after adjustment for weight change. In addition, high consumption of simple sugar was positively associated with an increased risk of MetS, which was independent of weight change. Moreover, refined grains intake was positively associated with the risk of MetS, and this association remained significant after adjustment for weight change. A lower risk of MetS was found among participants who experienced weight loss and consumed lower refined grains. However, among individuals with weight gain, consumption of refined grains, more than 6.6 servings/day, increased the risk of MetS.

In the current study, no associations between carbohydrates and MetS risk were identified. Several systematic reviews and meta-analyses of observational studies have aimed to synchronize evidence regarding total or specific carbohydrates with the risk of MetS [4], and other cardiometabolic diseases, including obesity, T2DM, and CVD [5, 37–39], yet the results have been conflicting. As indicated by a recent systematic review and meta-analysis, each 5% increase in carbohydrate intake increased the risk of MetS by 2.6%. It is worth mentioning that in the aforementioned meta-analysis, the heterogeneity between the studies was high (*I2* = 82.0%, P = 0.000), that might be in part due to the geographical location of studies [4]. Most observational studies in this meta-analysis were conducted in East Asia (9 of 19 studies), and a distinct difference exists in the macronutrient intake between the Western and East Asian populations. For example, based on the data from the U.S. National Health and Nutrition Examination Survey (NHANES) and the Korean version of NHANES (KNHAES), the proportion of energy from carbohydrates in the typical Korean diet (80–82%) was higher than that of in the western diet (64–65%) [40]. Notably, the threshold of carbohydrate intake for the management of MetS has been reported at 230 g [41], and carbohydrates intake ≥ 60% of total energy intake is a dietary risk factor for MetS [41]. Nationally representative data from the U.S. and Korea have indicated a positive association of higher dietary carbohydrate intake with odds of MetS in Koreans, but not Americans [40]. Moreover, results from the PURE, a large epidemiological cohort study including 18 countries, reported that higher carbohydrate intake was not associated with cardiovascular disease risk [8]. We also found no association between carbohydrate intake and MetS risk. In the current study, the carbohydrate intake (median, 53.1; IQR 46.0-58.6) was less than the upper recommended limit of carbohydrate intake (65%).

In the current study, the positive association between refined grains and the risk of MetS may reflect the amount of daily refined grains intake (6.6, IQR: 3.9–8.6) in which the risk of MetS increased at 6.6 servings/day. This is in line with previous systematic reviews and meta-analyses that reported consumption of refined grains at 200–400 g/day increased the risk of T2DM by 6–14% [42]. Globally, Food-Based Dietary Guidelines (FBDGs) suggest replacing refined grains with whole grains to promote healthy dietary patterns and reduce the risk of chronic diseases [43]. Similarly, a recently published systematic review and meta-analysis of 25 randomized control trials found that substituting refined grains for whole grains was associated with substantial improvements in MetS components [44]. However, The 2015 Dietary Guidelines Advisory Committee (DGAC), which informs the corresponding 2015–2020 Dietary Guidelines for Americans, reported that only half of the grains intake should be from whole grains and the rest can be obtained from refined grains [45]. Consumption of refined grains up to a certain threshold, 220 g/d in the Chinese population had no adverse health outcome [41]. Moreover, over 9 years of follow-up, consumption of refined grains with a mean intake of 2.87 serving/day in men and 2.07 serving/day in women was not associated with the risk of MetS in Atherosclerosis Risk in Communities (ARIC) [46]. However refined grains consumption in countries with typical diets, high in carbohydrates especially refined sources [8], is positively associated with the risk of MetS [47].

The World Health Organization has recommended limiting energy intake to 5% of simple sugar to prevent nutrient dilution in the diet [48]. Regarding the potentially detrimental effects of sugar in causing diet-related chronic diseases, the American Heart Association, The Institute of Medicine Carbohydrate, and Dietary Guidelines Advisory Committee 2015 have an reported an upper limit of 10 to 25% of energy intake [49]. Moreover, in modeling diets, a restricted intake of sugar between 5 and 10% of energy was recommended [50]. Our findings are inconsistent with the findings of prospective studies that reported no association between sucrose and T2DM risk during a 6-year follow-up [13–15]. In a clinical trial, consumption of 45 g of sucrose over a 6-week had no detrimental effect on glycemic control in participants with T2DM [51]. However, in another trial, recommendation on lowering the consumption of simple sugar and starchy carbohydrates improved glycemic control in participants with prediabetes and T2DM [52]. More studies are needed to confirm the relationship between simple sugar intake and MetS and the recommended percentage for the prevention of MetS. We also found that weight change did not modify the association between simple sugar and the risk of MetS. Participants with higher a simple sugar intake, regardless of their weight status, had a higher risk of MetS. Weight gain is a risk factor for MetS [18] and has been shown to modify the association between some dietary food groups such as fruits and vegetables and fruit juice [19, 20]. However, other studies have shown restriction of carbohydrate intake negative [21], and SSBs positively [20] associated with MetS, independent of weight changes. The effect modification of weight change on the association between a simple sugar and the risk of MetS and the appropriate percentage of sugar consumption for the prevention and management of MetS needs to be studied.

Evidence from systematic reviews and meta-analysis of observational studies and human trials suggest that whole grain intake is inversely associated with the risk of MetS [11], and related cardiometabolic outcomes [6, 10, 12]. With regards to whole grains intake, results from the current study corroborate with some [53], but not all the previous population-based cohort studies [54, 55]. In our total population, whole grains were negatively associated with the risk of MetS However, this association disappeared after adjustment for weight changes. After classifying participants based on weight changes, the risk of MetS decreased among subjects with weight loss, independent of whole grain intake. The inconsistency in results between whole grains intake and risk of MetS may be due to the heterogeneous associations of individual whole grain foods with the risk of chronic disease that has been previously reported [54]. The nutrient and phytochemical content, including fiber, magnesium, and phenolic compounds [56], in addition to the glycemic properties of individual whole grain foods, vary to a great extent [57], thereby influencing the potential favorable effects of whole grains on preventing chronic disease. Interestingly, recent findings from the China Nutrition and Health Database indicated that dietary fiber from whole grains is not associated with the risk of obesity, T2DM, and CVD, and suggested that the impact of whole grains may be overestimated among the Chinese population [58].

MetS is a carbohydrate intolerance state. In a meta-analysis of randomized controlled trials, a low carbohydrate diet was an effective diet in inducing weight loss, and improved dyslipidemia in MetS (high TG and low HDl-c) [59]. However separating the effects of a low carbohydrate diet from weight loss on cardiometabolic risk factors is challenging, as both influence each other and have a favorable effect on MetS and its components [60, 61]. In a randomized control trial, a low carbohydrate diet, independent of weight loss, reverses MetS and improved atherogenic dyslipidemia [21]. In contradiction with these findings, we found that in weight loss status, only consumption of carbohydrates less than 53.0% of total energy, reduces the risk of metabolic syndrome. Therefore, whether weight loss modulates the effects of carbohydrate restriction on the MetS and its components needs to be further investigated.

This study has several strengths. Dietary intake was assessed using a valid and reliable FFQ, a gold standard tool in assessing habitual dietary intake. Moreover, we used the alternative approach for the assessing of dietary intake which aims to reduce within-subject variability and evaluate the long-term diet more concisely. Additionally, the investigation of the effect modification of weight change on the association between carbohydrate intake and MetS risk across 8.9 years of follow-up were some of the important strengths of the current study. Besides, by conducting this study in the Middle East and North Africa region with different dietary habits than Western and Eastern countries, we can broaden our knowledge about overall carbohydrates and source of carbohydrate intake. However, the generalizability of our findings needs to be done with caution to other population because the association between carbohydrate intake and chronic disease differs according to ethnicity [25] and dietary habits [35, 36]. Another limitation of our study is that the potential of residual or unmeasured confounders cannot be ruled out. Moreover, because our study is observational, we are not able to establish causality. Finally, the present findings were based on 8.9 years of follow-up; prospective studies with a long follow-up period are needed to substantiate our conclusions.

## Conclusion

Our findings suggest an effect modification by weight change on the association between carbohydrates, and refined grains intake and the risk of MetS. Weight loss along with lower consumption of carbohydrates, and refined grains reduced the risk of MetS. However, simple sugar intake, regardless of weight change, was associated with an increased risk of MetS.

## Data Availability

The datasets generated and/or analyzed during the current study are not publicly available due institution’s policy but are available from the corresponding author on reasonable request.

## List of abbreviation

TLGS: Tehran Lipid and Glucose Study
MetS: metabolic syndrome
HRs: hazard ratios
ARIC: Atherosclerosis Risk in Communities
PURE: Prospective Urban Rural Epidemiology
T2DM: type 2 diabetes mellitus
CVD: cardiovascular disease
SSBs: sugar-sweetened beverages
TLGS: Tehran Lipid and Glucose Study
FFQ: food frequency questionnaire
RIES: Research Institute for Endocrine Sciences
BMI: body mass index
WC: waist circumference
MAQ: Modifiable Activity Questionnaire
MET: metabolic-equivalent
FCT: food composition table
FPG: fasting plasma glucose
HDL-C: high-density lipoprotein-cholesterol
TG: triglyceride
CV: coefficients of variation
NHANES: National Health and Nutrition Examination Survey
FBDGs: Food-Based Dietary Guidelines
DGAC: Dietary Guidelines Advisory Committee
ARIC: Atherosclerosis Risk in Communities
HEI: healthy eating index
